# Acral Melanoma in Chinese: A Clinicopathological and Prognostic Study of 142 cases

**DOI:** 10.1038/srep31432

**Published:** 2016-08-22

**Authors:** Jiaojie Lv, Bo Dai, Yunyi Kong, Xuxia Shen, Jincheng Kong

**Affiliations:** 1 Department of Pathology, Fudan University Shanghai Cancer Center, Shanghai, People’s Republic of China; 2 Department of Oncology, Shanghai Medical College, Fudan University, Shanghai, People’s Republic of China; 3 Department of Urology, Fudan University Shanghai Cancer Center, Shanghai, People’s Republic of China; 4 Department of Pathology, First People’s Hospital, Shanghai Jiaotong University, People’s Republic of China

## Abstract

Acral melanoma (AM), as a peculiar subgroup of melanoma, is rare in Caucasians but has higher incidence in Asians. Large series of study on AM with clinicopathological features and prognostic factors is still limited, especially in Asian population. We retrospectively collected clinical, pathological and follow-up data of 142 AM cases. All patients were Chinese, with the age ranging from 24 to 87 years (mean 62.0; median 62.0). The Breslow thickness of primary lesions ranged from 0.6 to 16.3 mm (mean 4.9; median 3.7). 85.9% of the patients had acral lentiginous histologic subtype. Plantar was the most frequently involved site, followed by heels. Statistically, duration of the lesion before diagnosis (≤2.5 years), Breslow thickness >4.0 mm (T4), high mitotic index (>15 mm^−2^), presence of vascular invasion, regional lymph node metastasis at diagnosis and pathologic stage (II/III/IV) were found to be independent prognostic factors in both univariate and multivariate analyses. The prognosis of AM in Chinese is extremely poor. Our 5- and 10-year disease-specific survival (DSS) rates were 53.3% and 27.4%, respectively. Therefore, AM in Asians represents a more biologically aggressive melanoma subtype and is thought to carry a worse prognosis when compared with other races or cutaneous melanomas in other anatomic sites.

Malignant melanoma is the most lethal form of skin cancer and its incidence has considerably increased in recent decades in most countries. Based on the anatomical location and the degree of sun exposure, melanoma is classified into 4 major subtypes: melanoma on skin without chronic sun-induced damage, melanoma on skin with chronic sun-induced damage, mucosal melanoma, and acral melanoma (AM)^1^. The Surveillance, Epidemiology, and End Results (SEER) data exhibited that the clinical features, anatomical origin, and patients’ prognoses differ significantly among different ethnic groups^2^. AM is rare in Caucasians (1–7%) but has higher incidence in non-White individuals^3^,^4^,^5^,^6^,^7^,^8^, accounting for up to 58% of all cutaneous melanomas in Asians^9^ and even more (60–70%) in Blacks^10^. It is often misdiagnosed or ignored before an accurate diagnosis is made because it is hidden from view^11^,^12^. Furthermore, AM may represent a more biologically aggressive melanoma subtype and is thought to carry a worse prognosis when compared with other melanoma subtypes^12^,^13^,^14^,^15^,^16^. However, only a few case series of AM has been published in Asia^17^,^18^ and knowledge on its characteristics and prognosis factors is still limited, especially in China which has the largest Asian population in the world. Herein, we present a large single-institution series of 142 patients with AM to identify clinical and pathological prognostic factors associated with disease-specific survival (DSS) for this peculiar subtype of melanoma.

## Results

### Clinical features

The clinical characteristics of the patients are summarized in Table 1. All the 142 patients were Chinese. There were 84 (59.2%) male patients and 58 (40.8%) female patients, with a male to female ratio of 1:0.69. The age of the patients at diagnosis ranged from 24 to 87 years (mean 62.0; median 62.0). Among 9 (6.3%) patients who recalled their exact trauma history, 5 were on the plantars, 4 were on the dorsum of the feet. Most trauma was caused by a mild injury, such as tearing on a stone, pricking on a spike or glasses, or stepping on charcoal. The diameter of the lesion, in terms of major axis, ranged from 0.3 to 7.5 cm (mean 2.5; median 2.0). The duration of the lesion before diagnosis was varied from 10 days to 70 years (mean 5.1 years; median 1.8 years).

In the nonungual area, the plantar was the most common region. We did not find any case on the palms or the dorsum of the hands. In the subungual sites, the toenail is more frequently involved than the fingernail. The commonest location of subungual melanoma (SUM) was the great toe [13 lesions (56.5%)] followed by the thumb [5 lesions (21.7%)]. The lesions were distributed approximately equally between the right and the left side.

**Figure 1:**
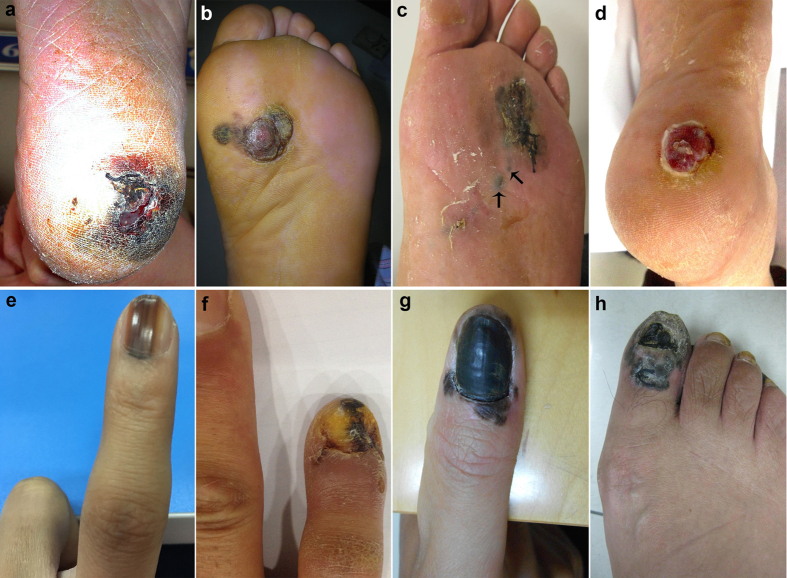
(**a**) AM on the heel, showing a rapidly expanding plaque with irregular margins and ulceration. (**b**) AM on the plantar, the tumorigenic vertical growth phase nodule is present within a background pigmented macule. (**c**) AM on the plantar, showing foci of satellites (arrows) discontinuous from the main irregular and pigmented lesion. (**d**) Achromic melanoma on the heel resembling granuloma pyogenium. (**e**) SUM showing pigmented strip on the index finger. (**f**) SUM on the little finger, thickening, splitting and destruction of the nail plate. (**g**) SUM on the thumb, spreading to the skin of the digit proximally, laterally, and distally (Hutchinson sign). (**h**) SUM on the great toe, the entire nail matrix and nail bed are occupied by irregular pigmented mass involving the ungual fold and periungual skin.

Manifestations of AM had a wide clinical spectrum. Lesions initially appeared as a pigmented macule, then progressed to a rapidly expanding plaque with irregular, notched borders, sometimes with ulceration (Fig. 1a). With the evolution of apparent vertical growth phase, an elevated papule or nodule developed within a background pigmented macule (Fig. 1b). Occasionally, multiple foci of satellites discontinuous from the main tumour were observed (Fig. 1c). In a minority of cases, it presented as achromic melanoma clinically resembling granuloma pyogenium (Fig. 1d). SUMs often began as brown to black discolouration of the nail that frequently became a well demarcated, pigmented longitudinal streak. With the time, the strip became bigger with indistinct, blurred margins (Fig. 1e). Thickening, splitting, or destruction of the nail plate may occur (Fig. 1f). The irregular macular hyperpigmentation can also spread to involve the skin of the digit, proximally, laterally and distally (Hutchinson sign, Fig. 1g). Eventually, the entire nail matrix and nail bed were occupied by a tan to black irregular plaque or mass, which involved the ungula fold and periungual skin (Fig. 1h).

### Histologic features and pathologic staging

**Figure 2:**
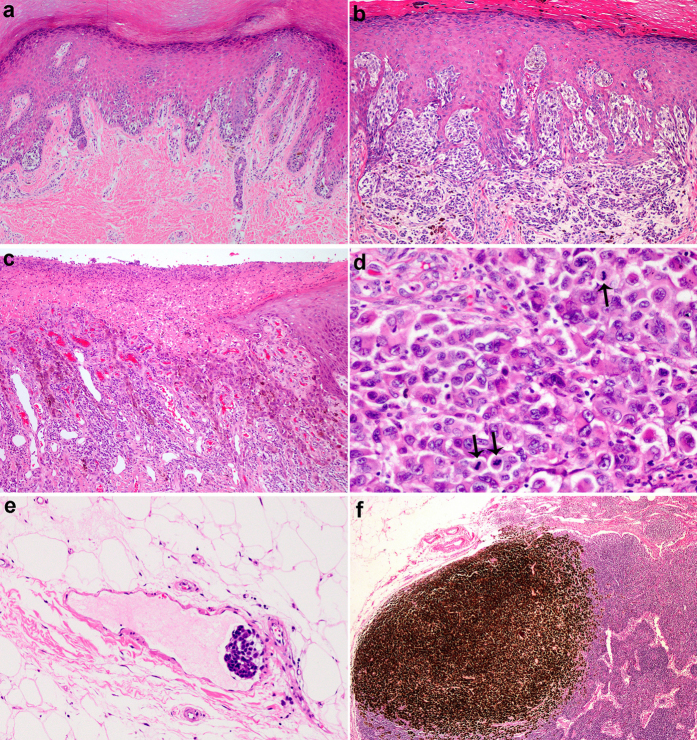
(**a**) ALM *in situ*. Lentiginous proliferation of atypical melanocytes along the basal epidermis (original magnification ×100). (**b**) ALM with vertical growth phase, Clark level IV (original magnification ×100). (**c**) Ulceration (original magnification ×100). (**d**) Scattered mitoses in dermal lesion of melanoma (arrows) (original magnification ×400). (**e**) Vascular invasion by melanoma cells in septa of adipose tissue (original magnification ×200). (**f**) Melanoma metastasis with prominent pigment in regional lymph node (original magnification ×40).

Histologic examination was performed on 142 AM patients by wide surgical excision. Two patients (1.4%) had a personal history of a noncutaneous cancer (one liver, one pancreas). The Breslow thickness of the primary lesion ranged from 0.6 to 16.3 mm (mean 4.9; median 3.7). Of the 142 cases, the most common histologic subtype of patients were acral lentiginous melanoma (ALM) [122 cases (85.9%)] followed by nodular melanoma (NM) [17 cases (12.0%)] and superficial spreading melanoma (SSM) [3 cases (2.1%)]. Pathological characteristics, including Breslow thickness, Clark level, ulceration, mitotic rate, tumour-infiltrating lymphocytes (TILs), vascular invasion, regional lymph node metastasis at diagnosis, and pathologic stage, are shown in Table 2. Some histologic characteristics of melanoma are shown in Fig. 2.

### Follow-up and survival analysis

The follow-up ranged from 5 to 151 months (mean 58.9; median 53.5). At the end of the study, 83 (58.5%) patients died of evolution of their melanoma, due to regional lymph node metastasis, in transit metastasis and/or distant metastasis (in brain, lung, liver, bone, bladder). 10 (7.0%) died of unrelated disease. 33 (23.2%) are alive without evidence of residual disease and 16 (11.3%) patients are alive after recurrence. The 5- and 10-year DSS rates were 53.3% and 27.4%, respectively. The 5-year DSS rates according to Breslow thickness are shown in Table 3.

**Figure 3:**
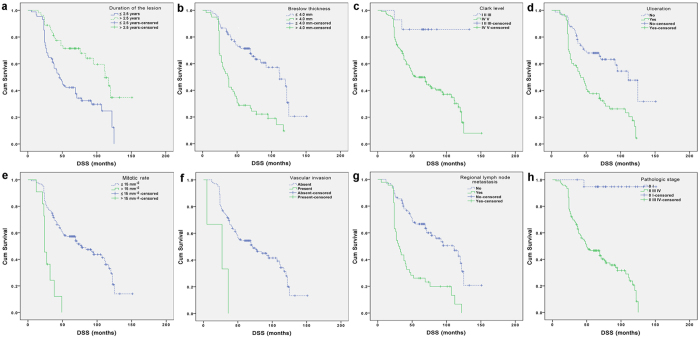
Kaplan-Meier analyses of DSS for the entire group of patients according to different stratums by prognostic factors. (**a**) Duration of the lesion. (**b**) Breslow thickness. (**c**) Clark level. (**d**) Ulceration status. (**e**) Mitotic rate. (**f**) Vascular invasion. (**g**) Regional lymph node status at diagnosis. (**h**) Pathologic stage.

Univariate analysis revealed that patients with one of the following poor prognostic factors (Table 4): duration of the lesion before diagnosis (≤2.5 years), Breslow thickness >4.0 mm (T4), Clark level (IV/V), presence of ulceration, high mitotic rate (>15 mm^−2^), presence of vascular invasion, regional lymph node metastasis at diagnosis, pathologic stage (II/III/IV) had significantly lower DSS (Table 4, Fig. 3a–h). Then, the eight significant factors in univariate analysis were introduced in a multivariate Cox model to identify independent prognostic factors. Duration of the lesion before diagnosis (≤2.5 years), Breslow thickness >4.0 mm (T4), high mitotic rate (>15 mm^−2^), presence of vascular invasion, regional lymph node metastasis at diagnosis and pathologic stage (II/III/IV) were found to be independent prognostic factors for DSS (Table 5).

## Discussion

The present study is one of the largest single-institution series of AM with a complete review of the histological slides and with thorough follow-up of the patients in Asia. There was no significant sex predominance in our study, which is similar to the results in most previous studies^12^,^13^,^14^,^16^,^17^,^18^,^19^. However, a few studies of Whites reported a clear ALM predominance in females^4^,^20^. The mean age of our cohort was 62.0 years, with a peak incidence during the sixth decade (51–60 years). The mean age is comparable with data from other studies (55–63 years)^4^,^12^,^13^,^14^,^16^,^18^,^20^, while the peak age is slightly younger than that in most studies (61–70 or ≥70 years)^14^,^18^,^20^ of both Caucasians and Asians. Plantar was indicated as most frequently involved site in AM^12^,^14^,^17^,^20^. In accordance with previous reports, most AM in our series arose on the feet, most frequently on plantar sites (41.6%), followed by heels (26.8%). In our study, SUM was more frequent on toes (11.3%) than on fingers (4.9%). However, previous studies focusing on SUM indicated that it occurred more often on fingers than toes^12^,^14^,^17^,^18^,^20^,^21^. This could be due to our few cases of SUM.

Long delay with a duration ranging from 1 to 3.7 years in diagnosis of AM was described in the most representative reports^19^,^22^,^23^,^24^,^25^. Many factors seem to contribute to the postponement of diagnosis: elder patients, hidden site, frequent lack of pigmentation, lack of recognition and misdiagnosis by dermatologists sometimes. Clinical differential diagnoses for nonungual AM cases include wart, callus, fungal disorder, pyogenic granuloma *et al*. SUMs are often mistaken for chronic paronychia, subungual haematoma, keratoacanthoma, nonhealing ulcer, tinea *et al*. Interestingly and notably, our study demonstrated that patients with duration of the lesion before diagnosis (≤2.5 years) had significantly lower DSS. We considered that the tumour of shorter disease course may progress more rapidly and tends to be more aggressive than that of longer disease duration.

In the largest population-based evaluation of acral-lentiginous melanoma by Bradford *et al*., Asia/Pacific Islanders had the highest percentage of T4 compared to Whites and Blacks^13^. In consistent with previous data, our study indicated a high proportion of Breslow thickness of T4 (40.8%) and it is even higher than that reported in Koreans (33%)^18^. In addition, the mean Breslow thickness in our study was 4.9 mm, which is much more thicker than that in studies of Whites (2.0–2.6 mm)^12^,^14^,^16^,^26^. Therefore, we conclude that AM had a more advanced thickness in Asians, especially in Chinese. Furthermore, Breslow thickness (>4.0 mm) was a significant prognostic factor in both univariate and multivariate DSS analyses in our study. It was also indicated as an independent adverse prognostic factor in three of the four studies evaluating disease-free survival (DFS)^12^,^20^,^27^,^28^, and in three of the five studies evaluating overall survival (OS) or DSS^12^,^16^,^20^,^29^,^30^.

The proportion of ulceration (47.9%) in our series was higher than that previously described in both Asians and Whites (36–42%)^4^,^14^,^16^,^18^. In our study, ulceration was significantly associated with a lower DSS in univariate analysis, but did not show any significant effect in multivariate analysis. Other studies indicated that it has an independent prognostic value in one of the two studies evaluating DFS^12^,^27^ and in three of the four studies evaluating OS^12^,^18^,^30^ or DSS^16^. In addition, another prominent finding in our study was that high mitotic rate (>15 mm^−2^) appeared to be a powerful independent prognostic factor. The mitotic rate reflects the proliferative activity in a neoplastic system. There was only a few reports of AM that had done some research on it^16^,^18^,^26^. Only mitotic rate >6.0 mm^−2^ was found to be associated with a greater relative risk for short DFS and DSS in ALM study of Whites by Phan *et al*.^26^.

The prognosis of Chinese patients with AM was clearly not optimistic: The 5- and 10-year DSS rates in our patients were 53.3% and 27.4%, respectively. In the largest report of AM, Bradford *et al*. reported a population-based analysis, with 5- and 10-year DSS rates of 80.3% and 67.5%, respectively^13^. They also concluded that 5- and 10-year DSS rates were highest in non-Hispanic Whites (82.6% and 69.4%), intermediate in Blacks (77.2% and 71.5%), and lowest in Hispanic Whites (72.8% and 57.3%) and Asian/Pacific Islanders (70.2% and 54.1%). Bello *et al*. reported a 5-year DSS rate of 70%^16^, comparable with the rate reported by Kuchelmeister *et al*. (71%)^14^ and Phan *et al*. (76%)^20^. Our 5-year DSS results are substantially worse than those observed in AM (70–82%)^14^,^16^ and ALM (76–80%)^19^,^20^,^26^ of Whites. However, our data are comparable with those observed in other Asian studies. 5-year survival rates of AM reported from Korean and Japan ranged from 35.0% to 49.3%^17^,^18^. In addition, when controlling the survival rates for Breslow thickness, our 5-year DSS rates of AM were still 10–30% lower than those in previous studies^13^,^19^. So we concluded that the DSS rates of AM in Asians are worse than that in other races. Several factors including high ulcerative rate (47.9%), extreme proportion of Breslow thickness >4 mm (40.8%), high mitotic rate (>15 mm^−2^) may contribute to the poor survival in our patients. In addition, lack of recognition by patients and misdiagnosed as benign by clinicians sometimes can also contribute to the poor survival in our patients.

In conclusion, this study represents one of the largest single-institution series describing the clinicopathological characteristics and prognostic factors of AM in Asia. Duration of the lesion before diagnosis (≤2.5 years), Breslow thickness >4.0 mm (T4), Clark level (IV/V), presence of ulceration, high mitotic rate (>15 mm^−2^), presence of vascular invasion, regional lymph node metastasis at diagnosis and pathologic stage (II/III/IV) are prognostic indicators associated with DSS. As a particular subgroup of melanoma, the prognosis of AM in Asians is worse, compared with AM in other ethnic group and cutaneous melanomas in other sites.

## Methods

### Clinical data

The computerized databases in the pathology department of Fudan University Shanghai Cancer Center were used for the study. The key words ‘melanoma’ were used to identify the patients. Among them, melanomas located on acral sites were included. Data were collected between January 2004 and December 2010 to allow determination of 5-year survival statistics. Only those patients with primary tumour and treated with wide surgical excision at our institution were included in our study. After reviewing the clinical records, pathological slides and complete follow-up data of these patients, 142 patients with AM were included in the present study. Informed consent was obtained from each patient. The study has been approved by Institutional Ethics Committee at Fudan University Shanghai Cancer Center. All procedures were conducted according to the guidelines approved by Institutional Ethics Committee at Fudan University Shanghai Cancer Center.

For each patient, the following clinical data were retrieved from medical records: sex, age at the time of diagnosis, the presence or absence of a history of trauma, longest diameter of lesion, duration of the lesion, and the site of lesion. The duration of the lesion was recorded as the time between the first notice by the patient of an abnormality and surgery-proven diagnosis. In case of incomplete or unavailable information, telephone interviews of the patients or of their families were systematically used. Clinical description of the primary lesion was based on clinical records and review of clinical preoperative photographs. For each patient, information on follow-up was recorded: clinical status at the latest contact (alive without evidence of recurrence, alive after recurrence, dead of the disease, dead of another disease). In addition, survival curves were calculated based on the medical records of patients who died of melanoma.

### Histopathological study and pathologic staging

Pathological slides of surgical specimens were reviewed by two experienced dermatopathologists. The following data were recorded: histologic subtype, Breslow thickness, Clark level, ulceration, mitotic rate (mitotic count per millimeters squared), TILs, presence of vascular invasion, regional lymph node metastasis at diagnosis. The pathologic stage of disease was determined based on the most recent classification of the American Joint Committee on Cancer (AJCC)^31^.

### Statistical analysis

The evaluation of data was performed using the statistical package SPSS, version 22.0 (SPSS, Inc). The Kaplan-Meier method was used to calculate survival curves, and significant differences were determined by the log-rank test. DSS was defined as the time from pathological diagnosis to the time of death due to melanoma or last follow-up. Univariate Cox regression model was used to examine the association of clinical and pathologic variables with DSS. Characteristics significant on univariate analysis with a p value of <0.05 were entered into a multivariate Cox proportional hazards model.

## Additional Information

**How to cite this article**: Lv, J. *et al*. Acral Melanoma in Chinese: A Clinicopathological and Prognostic Study of 142 cases. *Sci. Rep.* **6**, 31432; doi: 10.1038/srep31432 (2016).

## Figures and Tables

**Table 1:** Clinical characteristics of Chinese patients with acral melanoma.

Characteristics	No. (%)
Sex	
Male	84 (59.2)
Female	58 (40.8)
Age, years	
<31	3 (2.1)
31–40	4 (2.8)
41–50	18 (12.7)
51–60	37 (26.1)
61–70	36 (25.4)
71–80	34 (23.9)
>80	10 (7.0)
Trauma history	
Yes	9 (6.3)
No	133 (93.7)
Longest diameter of lesion, cm	
<1.0	8 (5.6)
1.0–2.0	64 (45.1)
2.0–3.0	37 (26.1)
3.0–4.0	21 (14.8)
4.0–5.0	6 (4.2)
>5.0	6 (4.2)
Duration of the lesion, years	
<1	45 (31.7)
1–2.5	43 (30.3)
2.5–5	24 (16.9)
5–7.5	4 (2.8)
7.5–10	5 (3.5)
>10	21 (14.8)
Site of lesion	
Nonungual location	119 (83.8)
Finger	4 (2.8)
Toe	10 (7.0)
Foot	105 (74.0)
Plantar	59 (41.6)
Heel	38 (26.8)
Dorsum	8 (5.6)
Subungual location	23 (16.2)
Fingernail	7 (4.9)
Toenail	16 (11.3)

**Table 2:** Pathological characteristics of Chinese patients with acral melanoma.

Characteristics	No. (%)
Histologic subtype	
ALM	122 (85.9)
NM	17 (12.0)
SSM	3 (2.1)
Breslow thickness, mm	
≤1.0	8 (5.6)
1.01–2.0	21 (14.8)
2.01–4.0	55 (38.8)
>4.0	58 (40.8)
Clark level	
I	1 (0.7)
II	10 (7.0)
III	3 (2.1)
IV	83 (58.5)
V	45 (31.7)
Ulceration	
Yes	68 (47.9)
No	74 (52.1)
Mitotic rate, mm^−2^	
≤15	131 (92.3)
>15	11 (7.7)
TILs	
Absent	46 (32.4)
Brisk	9 (6.3)
Non-brisk	87 (61.3)
Vascular invasion	
Absent	139 (97.9)
Present	3 (2.1)
Regional lymph node metastasis at diagnosis	
Yes	46 (32.4)
No	96 (67.6)
Pathologic stage	
0	1 (0.7)
I	19 (13.4)
II	74 (52.1)
III	44 (31.0)
IV	4 (2.8)

ALM, acral lentiginous melanoma; NM, nodular melanoma; SSM, superficial spreading melanoma; TILS, tumour-infiltrating lymphocytes.

**Table 3:** Specific survival rates according to Breslow thickness.

Overall	5 year survival (%)
Thickness ≤1.0 mm	100
Thickness 1.01–2.0 mm	66.7
Thickness 2.01–4.0 mm	56.4
Thickness >4.0 mm	27.6

**Table 4:** Univariate analysis of factors associated with disease-specific survival in the acral melanoma cohort.

Characteristics	No. Pts	No. of DOD (%)	P value*
All patients	142	83 (58.5)	
Sex			
Male	84	48 (57.1)	
Female	58	35 (60.3)	0.512
Age, years			
<55	41	26 (63.4)	
≥55	101	57 (56.4)	0.732
Trauma history			
Yes	8	5 (62.5)	
No	134	78 (58.2)	0.486
Longest diameter of lesion, cm			
≤2	72	42 (58.3)	
>2	70	41 (58.6)	0.492
Duration of the lesion, years			
≤2.5	88	61 (69.3)	
>2.5	54	22 (40.7)	<0.001
Site of lesion			
Nonungual location	119	68 (57.1)	
Subungual location	23	15 (65.2)	0.895
Histologic subtype			
ALM	122	67 (54.9)	
Other	20	16 (80.0)	0.081
Breslow thickness, mm			
≤4.0	83	35 (42.2)	
>4.0	59	48 (81.4)	<0.001
Clark level			
I/II/III	14	2 (14.3)	
IV/V	128	81 (63.3)	0.009
Ulceration			
Yes	68	54 (79.4)	
No	74	29 (39.2)	<0.001
Mitotic rate, mm^−2^			
≤15	131	73 (55.7)	
>15	11	10 (90.9)	<0.001
TILs			
Absent	46	21 (45.7)	
Present	96	62 (64.6)	0.155
Vascular invasion			
Absent	139	80 (57.6)	
Present	3	3 (100.0)	0.002
Regional lymph node metastasis at diagnosis			
Yes	46	39 (84.8)	
No	96	44 (45.8)	<0.001
Pathologic stage			
0/I	20	1 (5.0)	
II/III/IV	122	82 (67.2)	<0.001

^*^Log-rank test; DOD, died of disease.

**Table 5:** Multivariate analysis of factors associated with disease-specific survival in the acral melanoma cohort.

Characteristics	HR (95% CI)	P value
Duration of the lesion (≤2.5 vs >2.5)	0.056 (0.332–0.930)	0.025
Breslow thickness (≤4.0 vs >4.0)	1.749 (1.060–2.886)	0.029
Clark level (I II III vs IV V)	1.188 (0.276–5.113)	0.817
Ulceration (Yes vs No)	0.869 (0.524–1.442)	0.588
Mitotic rate (≤15 vs >15)	2.399 (1.146–5.019)	0.020
Vascular invasion (Absent vs Present)	0.272 (0.082–0.901)	0.033
Regional lymph node metastasis at diagnosis (Yes vs No)	1.757 (1.105–2.792)	0.017
Pathologic stage (0 I vs II III IV)	12.891 (1.689–98.425)	0.014

## References

[b1] Dai B. . Analysis of KIT expression and gene mutation in human acral melanoma: with a comparison between primary tumors and corresponding metastases/recurrences. Human pathology 44, 1472–1478 (2013). 23528861 10.1016/j.humpath.2013.01.007

[b2] Cormier J. N. . Ethnic differences among patients with cutaneous melanoma. Archives of internal medicine 166, 1907–1914 (2006). 17000949 10.1001/archinte.166.17.1907

[b3] Shaw J. H. & Koea J. B. Acral (volar-subungual) melanoma in Auckland, New Zealand. The British journal of surgery 75, 69–72 (1988). 3337957 10.1002/bjs.1800750125

[b4] Cascinelli N. . Acral lentiginous melanoma. A histological type without prognostic significance. The Journal of dermatologic surgery and oncology 20, 817–822 (1994). 7798414 10.1111/j.1524-4725.1994.tb03711.x

[b5] Ridgeway C. A., Hieken T. J., Ronan S. G., Kim D. K. & Das Gupta T. K. Acral lentiginous melanoma. Archives of surgery 130, 88–92 (1995). 7802583 10.1001/archsurg.1995.01430010090019

[b6] Richard M. A. . Delays in diagnosis and melanoma prognosis (I): the role of patients. International journal of cancer 89, 271–279 (2000). 10861504

[b7] Paladugu R. R., Winberg C. D. & Yonemoto R. H. Acral lentiginous melanoma. A clinicopathologic study of 36 patients. Cancer 52, 161–168 (1983). 6850538 10.1002/1097-0142(19830701)52:1<161::aid-cncr2820520129>3.0.co;2-r

[b8] Cress R. D. & Holly E. A. Incidence of cutaneous melanoma among non-Hispanic whites, Hispanics, Asians, and blacks: an analysis of california cancer registry data, 1988–93. Cancer causes & control 8, 246–252 (1997). 9134249 10.1023/a:1018432632528

[b9] Chang J. W. . Malignant melanoma in Taiwan: a prognostic study of 181 cases. Melanoma research 14, 537–541 (2004). 15577327 10.1097/00008390-200412000-00016

[b10] Hudson D. A. & Krige J. E. Melanoma in black South Africans. Journal of the American College of Surgeons 180, 65–71 (1995). 8000657

[b11] Gutman M. . Acral (volar-subungual) melanoma. The British journal of surgery 72, 610–613 (1985). 4027531 10.1002/bjs.1800720809

[b12] Slingluff C. L. Jr., Vollmer R. & Seigler H. F. Acral melanoma: a review of 185 patients with identification of prognostic variables. Journal of surgical oncology 45, 91–98 (1990). 2214797 10.1002/jso.2930450207

[b13] Bradford P. T., Goldstein A. M., McMaster M. L. & Tucker M. A. Acral lentiginous melanoma: incidence and survival patterns in the United States, 1986–2005. Archives of dermatology 145, 427–434 (2009). 19380664 10.1001/archdermatol.2008.609 PMC2735055

[b14] Kuchelmeister C., Schaumburg-Lever G. & Garbe C. Acral cutaneous melanoma in caucasians: clinical features, histopathology and prognosis in 112 patients. The British journal of dermatology 143, 275–280 (2000). 10951133 10.1046/j.1365-2133.2000.03651.x

[b15] Pollack L. A. . Melanoma survival in the United States, 1992 to 2005. Journal of the American Academy of Dermatology 65, S78–S86 (2011). 22018071 10.1016/j.jaad.2011.05.030 PMC4890628

[b16] Bello D. M. . Prognosis of acral melanoma: a series of 281 patients. Annals of surgical oncology 20, 3618–3625 (2013). 23838913 10.1245/s10434-013-3089-0

[b17] Seui M. . Acral melanoma in Japan. The Journal of investigative dermatology 80, 56s–60s (1983). 20479736 10.1038/jid.1983.15

[b18] Jung H. J., Kweon S. S., Lee J. B., Lee S. C. & Yun S. J. A clinicopathologic analysis of 177 acral melanomas in Koreans: relevance of spreading pattern and physical stress. JAMA dermatology 149, 1281–1288 (2013). 24067997 10.1001/jamadermatol.2013.5853

[b19] Boriani F., O’Leary F., Tohill M. & Orlando A. Acral Lentiginous Melanoma-misdiagnosis, referral delay and 5 years specific survival according to site. European review for medical and pharmacological sciences 18, 1990–1996 (2014). 25027337

[b20] Phan A. . Acral lentiginous melanoma: a clinicoprognostic study of 126 cases. The British journal of dermatology 155, 561–569 (2006). 16911282 10.1111/j.1365-2133.2006.07368.x

[b21] Tan K. B. . Subungual melanoma: a study of 124 cases highlighting features of early lesions, potential pitfalls in diagnosis, and guidelines for histologic reporting. The American journal of surgical pathology 31, 1902–1912 (2007). 18043047 10.1097/PAS.0b013e318073c600

[b22] Rigby H. S. & Briggs J. C. Subungual melanoma: a clinico-pathological study of 24 cases. British journal of plastic surgery 45, 275–278 (1992). 1623342 10.1016/0007-1226(92)90051-x

[b23] Kato T., Suetake T., Sugiyama Y., Tabata N. & Tagami H. Epidemiology and prognosis of subungual melanoma in 34 Japanese patients. The British journal of dermatology 134, 383–387 (1996). 8731657

[b24] Quinn M. J., Thompson J. E., Crotty K., McCarthy W. H. & Coates A. S. Subungual melanoma of the hand. The Journal of hand surgery 21, 506–511 (1996). 8724488 10.1016/S0363-5023(96)80371-6

[b25] Papachristou D. N. & Fortner J. G. Melanoma arising under the nail. Journal of surgical oncology 21, 219–222 (1982). 7144198 10.1002/jso.2930210405

[b26] Phan A. . Acral lentiginous melanoma: histopathological prognostic features of 121 cases. The British journal of dermatology 157, 311–318 (2007). 17596173 10.1111/j.1365-2133.2007.08031.x

[b27] Moehrle M. . “Functional” surgery in subungual melanoma. Dermatologic surgery 29, 366–374 (2003). 12656815 10.1046/j.1524-4725.2003.29087.x

[b28] Bormann G., Marsch W. C., Haerting J. & Helmbold P. Concomitant traumas influence prognosis in melanomas of the nail apparatus. The British journal of dermatology 155, 76–80 (2006). 16792755 10.1111/j.1365-2133.2006.07235.x

[b29] Barnes B. C., Seigler H. F., Saxby T. S., Kocher M. S. & Harrelson J. M. Melanoma of the foot. The Journal of bone and joint surgery. American volume 76, 892–898 (1994). 8200896 10.2106/00004623-199406000-00013

[b30] Heaton K. M., el-Naggar A., Ensign L. G., Ross M. I. & Balch C. M. Surgical management and prognostic factors in patients with subungual melanoma. Annals of surgery 219, 197–204 (1994). 8129491 10.1097/00000658-199402000-00012 PMC1243122

[b31] Balch C. M. . Final version of 2009 AJCC melanoma staging and classification. Journal of clinical oncology : official journal of the American Society of Clinical Oncology 27, 6199–6206 (2009). 19917835 10.1200/JCO.2009.23.4799 PMC2793035

